# Lipid recovery from a vegetable oil emulsion using microbial enrichment cultures

**DOI:** 10.1186/s13068-015-0228-9

**Published:** 2015-03-10

**Authors:** Jelmer Tamis, Dimitry Y Sorokin, Yang Jiang, Mark C M van Loosdrecht, Robbert Kleerebezem

**Affiliations:** Department of Biotechnology, Delft University of Technology, Julianalaan 67, 2628 BC Delft, The Netherlands; Winogradsky Institute of Microbiology, RAS, Leninskii avenue, 14, Leninskii avenue, 32а, Moscow, 119991 Russia

**Keywords:** Vegetable oil, Storage compounds, Microbial enrichment culture, Feast-famine, Resource recovery

## Abstract

**Background:**

Many waste streams have a relatively high vegetable oil content, which is a potential resource that should be recovered. Microbial storage compound production for the recovery of lipids from lipid-water emulsions with open (unsterilized) microbial cultures was investigated in a sequencing batch reactor using a diluted vegetable oil emulsion as model substrate.

**Results:**

After feeding, triacylglycerides (TAG) were accumulated intracellular by the microbial enrichment culture and subsequently used for growth in the remainder of the sequencing batch cycle. Roughly 50% of the added TAG could be recovered as intracellular lipids in this culture. The maximum lipid storage capacity of the enrichment culture was 54% on volatile suspended solids (VSS) mass basis in a separate fed-batch accumulation experiment. The microbial community was dominated by a lipolytic fungus, *Trichosporon gracile*, that was responsible for intracellular lipid accumulation but also a significant fraction of lipolytic and long chain fatty-acid-utilizing bacteria was present.

**Conclusion:**

Herewith, we demonstrate an effective strategy for enrichment of a microbial community that can accumulate significant amounts of lipids from wastewaters without the need for sterilization of substrates or equipment. Further optimization of this process will make recovery of lipids from wastewater possible.

**Electronic supplementary material:**

The online version of this article (doi:10.1186/s13068-015-0228-9) contains supplementary material, which is available to authorized users.

## Introduction

The use of agro-industrial organic residues for the production of valuable commodities is a logical step towards a bio-based economy. Traditionally, these wastes are used for biogas or compost production. Recently, several alternatives for the production of more valuable compounds have been proposed, for example, the production of biopolymers [1,2], volatile fatty acids [3], or medium chain length fatty acids [4]. One of the critical issues related to resource recovery from wastewater is the efficient up-concentration and purification of the valuable compounds that are present in diluted form in the wastewater. In general, the production of storage compounds with microbial cultures from diluted waste streams improves the efficiency of resource recovery since the product is concentrated inside the biomass and can be readily separated from the water using standard sludge settling/separation methods.

A relatively well studied example of a process based on storage compound production by microbial enrichment cultures is the production of polyhydroxyalkanoate (PHA) and contents up to 90% on VSS mass basis have been obtained by application of selective pressure in the form of feast-famine conditions [5,6]. This process is currently further evaluated in industrial environments [7-9]. Interestingly, it has been found that different carbon sources in a feast-famine process can result in formation of different storage compounds: microbial cultures enriched on glucose or starch, produced polyglucose as storage compound [10,11], a microbial culture enriched on glycerol produced a mixture of polyglucose and PHA [12], microbial communities enriched on different types of volatile fatty acids (VFA) produced different varieties of PHA [13,14], and a microbial culture enriched on methanol did not produce any storage compounds [15]. While the above examples illustrate the many substrate types that have been investigated, the fate of lipids as substrate in a feast-famine process remains unclear.

Lipids are an important constituent of many types of wastewater, especially triacylglycerides (TAG), present in (among others) effluents from vegetable oil crop processing industries. For example, in the wastewater from the palm oil industry, high concentrations of emulsified oil are found that are hard to recover using physical-chemical methods [16]. Palm oil is a fast growing market with a current production of around 50 Mton/year [17] accompanied by the production of 3 m^3^ wastewater per ton oil palm oil produced [18] resulting in an estimated palm oil mill effluent flow of 150 million m^3^/year.

It was proposed that lipids found in wastewater may be recovered through the use of lipid-accumulating microorganism for the production of biodiesel [19]. The economic feasibility of lipid extraction for diesel production from sludge will depend on the lipid content accumulated in the sludge but a cost of around 1 US $/l was reported from sludge containing 10% lipids [20]. In this approach, the recovery of lipids from wastewater requires a micro-organism with an as high TAG storage capacity as possible. However, not all microorganisms store TAG from wastewater in an appropriate way for biodiesel production.

**Figure 1:**
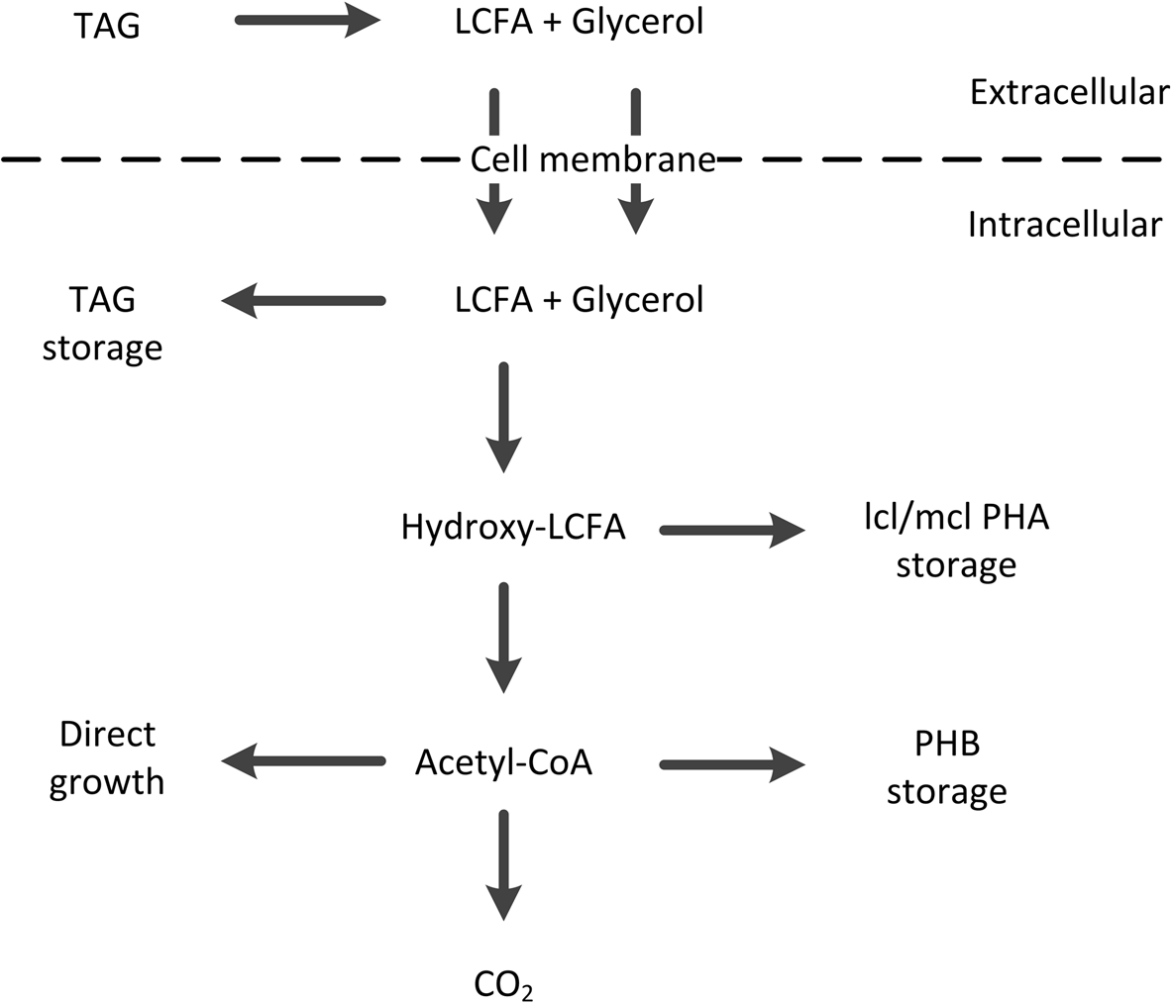
**Simplified metabolic pathways of storage compound formation and growth on TAG based on literature reports [** 21 **-** 29 **,** 38 **].** LCFA, long chain fatty acid; PHB, polyhydroxybutyrate; TAG, triacylglycerides.

A variety of metabolic pathways related to the microbial conversion of TAG have been described in literature (Figure 1). Due to the relatively large molecule size (M_w_ around 800 to 1,000 g/mol) and strongly hydrophobic nature of TAG, the uptake mechanism involves an extracellular hydrolysis step yielding long chain fatty acids (LCFA) and glycerol, which are subsequently transported into the cell [21]. Once the substrate is taken up, different routes are possible for the production of storage compounds: for example, PHA production by pure cultures of prokaryotic strains was reported [22-25] as well as the intracellular storage of TAG by pure cultures, for example, by oleaginous yeasts [26,27] or bacteria [28,29].

One approach for the recovery of lipids from wastewater is the cultivation of pure cultures of strains with very high lipid storage capacity. This approach has been evaluated in laboratory experiments with synthetic substrates and sterilized molasses [27]. However, since sterilization of diluted wastewater streams for biodiesel production is likely economically unfeasible, the use of unsterilized wastewater was evaluated [30,31]. In these cases, a pre-cultivated pure culture of oleaginous yeast was used to inoculate a batch experiment with acidic wastewater of a distillery as substrate. It was observed that a biomass with a relatively high lipid content (respectively 44% and 14%) could be grown by this method.

A different strategy was proposed by Santamauro *et al*. [32] in which an oleaginous yeast could be selectively enriched in a low pH environment. In general, oleaginous yeasts thrive relatively well in low pH environments. Nevertheless, many non-oleaginous microorganisms are known to be able to grow under low pH conditions [33]. The selective pressure imposed by the low pH (or temperature) does not intrinsically provide a competitive advantage for lipid-accumulating microorganisms, which makes the enrichment culture unstable and likely not optimal for lipid accumulation [34].

Since lipid accumulation has been reported as a microbial survival strategy to balance carbon and energy requirements in periods of absence of external substrate [35], in this study, we propose the application of a feast-famine strategy as a novel approach for the enrichment of lipid-accumulating organisms. This strategy is analogous to the feast-famine process for PHA production [6] and is based on ecological principles that provide an intrinsic competitive advantage to lipid-accumulating species. This process potentially enables the recovery of lipids from a broader spectrum of wastewaters, can be operated as sequenced batch or continuously, and alleviates the need for single batch operations with pre-cultivated pure culture inocula. An open (non-sterilized) reactor system was inoculated with wastewater sludge and pulse-fed with a model substrate (soybean oil) to evaluate the potential of the feast-famine principle for the recovery of lipids from diluted lipid-containing emulsions.

## Results

### Reactor operation

**Figure 2:**
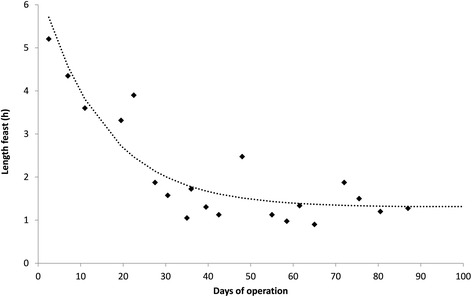
**Monitoring of the start-up of the sequencing batch reactor by the evolution of the length of the period needed for TAG substrate uptake (feast phase) by the enrichment culture in the reactor.** h, hours.

After inoculation of the sequencing batch reactor (SBR) with activated sludge (WWTP Dokhaven, The Netherlands) a typical feast-famine pattern became apparent in less than 5 days (10 cycles). The length of the feast phase (that is, time it took to take up the lipid substrate added with the influent) was initially more than 3 h and became more stable after 25 days (50 cycles) with a feast phase length of around 1 to 2 h (Figure 2). Variations in the length of the feast phase are due to the formation of biofilm in the reactor, which had to be periodically removed.

After the reactor had reached a stable operational performance, the carbon mass balance over the system was evaluated by analysis of the influent and effluent in the period of day 68 until day 76 of operation (16 cycles). The average effluent total suspended solids (TSS) concentration was 1.0 ± 0.16 g/l (standard deviation over the dataset of 16 cycles) with an ash fraction of 0.14 ± 0.03, resulting in a volatile suspended solids (VSS) concentration of 0.86 ± 0.15 g/l. Additionally, the amount of biofilm that was formed on the reactor wall was removed and measured, indicating an average biofilm growth of 0.25 gVSS per cycle. TOC measurements indicated that the carbon content of the organic solids in the effluent was 0.59 (±0.03) gC/gVSS resulting in a total organic solids production equivalent to 59 ± 10 Cmmol per cycle. Off-gas measurements showed a CO_2_ production of 17 ± 3 mmol per cycle. Herewith, most of the carbon present in the influent substrate (83 Cmmol/cycle) could be accounted for by carbon in the produced organic solids and CO_2_ (76 ± 10 Cmmol/cycle). The 10% deviation in the balance is partly due to the complications arising from the reactor wall growth.

**Figure 3:**
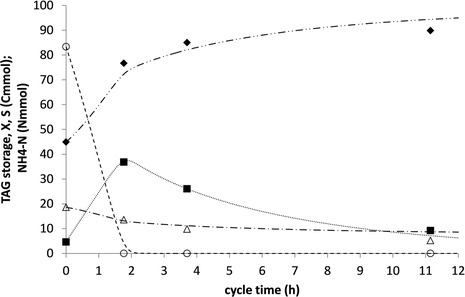
**Amounts of TAG substrate (○, −−−--), lipid storage (∎, ∙∙∙∙∙), active biomass (♦, −∙∙ − ∙∙−), and ammonium (∆, −∙ − ∙−) during a cycle in the TAG-fed sequencing batch reactor.** Symbols represent measurement and lines represent model-based calculations. h, hours; TAG, triacylglycerides.

To get further insight into the conversions during the cycle, three experiments were performed. For all three cycle experiments, the biomass concentrations and length of feast phase were within the range determined during the steady operational period. In Figure 3, profiles from a typical cycle are shown. For practical purposes and in order to establish the substrate amount accurately, the substrate in these experiments was dosed manually in one short pulse, and the amount of substrate was evaluated by the weight of the dosing syringe before and after addition of the oil.

**Figure 4:**
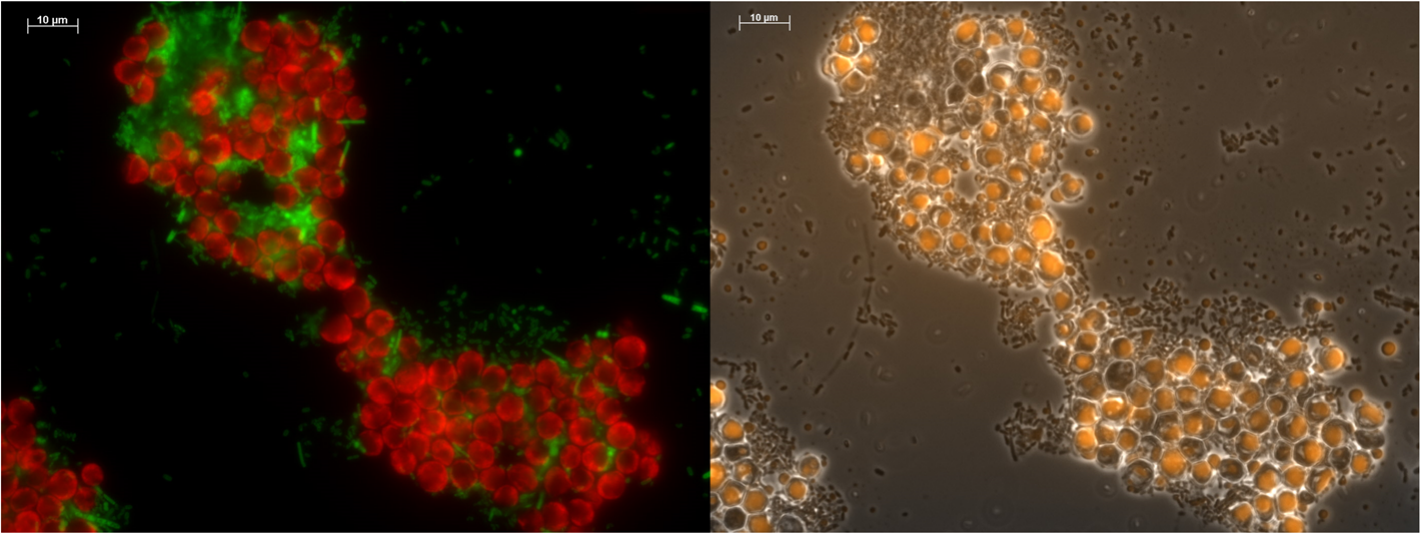
**Microscope images of a sample from the reactor at the end of the feast phase.** Left: FISH staining with the EUK516 probe to stain eukaryotes (red) and the EUB338 probe to stain prokaryotes (green). Right: Nile blue A staining to identify lipids (orange).

After dosing the vegetable oil, the reactor liquid became opaque, indicating that the mixture of microorganisms and vegetable oils formed an emulsion; this opaque feature disappeared again at the end of the feast phase (after 2 h) when the TAG was depleted from the medium. Gas chromatography (GC) analysis showed that the lipid content of the biomass increased from 6% ± 0.6% on VSS mass basis, at the start of the cycle, to 25% ± 1.5% on VSS mass basis at the end of the feast phase (average ± standard deviation over three cycle experiments), indicating significant storage of lipids (this was also confirmed by microscope images: Figure 4, right). The composition of the accumulated microbial lipids was almost identical to the substrate oil (Table 1), indicating direct utilization of the externally hydrolyzed LCFA and glycerol without their *de novo* synthesis or conversion. GC analysis showed only peaks that corresponded with the LCFA standard, and no significant peaks corresponding with the PHA standards, suggesting that TAG was the only relevant storage compound present in the culture at any time during the cycle. The lipid content of the cells decreased during the famine phase indicating growth on the stored lipids. Nitrogen was present throughout the cycle, and it was observed that nitrogen was taken up both during the feast and the famine phase, indicating significant microbial growth during the whole SBR cycle. The increase in VSS concentration in the feast phase was higher than that would be expected from TAG storage only, supporting the indication of growth of microorganisms in both the feast phase and the famine phase. A model was calibrated to the experimental data to identify characteristic process parameters (Additional file 1: Appendix C). It was estimated that about half of the substrate was used for growth while the other half was accumulated as intracellular lipids during the feast phase. An overall biomass-specific uptake rate of 1.0 Cmol/(Cmol h), a yield of lipid storage on substrate of 0.9 Cmol/Cmol and biomass on lipids of 0.6 to 0.7 Cmol/Cmol were estimated from the cycle measurements. A summary of the experimental data obtained during steady operation and cycle measurements in terms of carbon flows is provided in the (Additional file 1: Appendix B).

**Table 1:** **Comparison of the LCFA constituents from literature, used substrate and intracellular storage compounds**

	**Soybean oil [** 47 **]**	**Soybean oil (this study)**	**Intracellular storage (this study)**
	**Percentage of LCFA mass**		
Oleic acid	23%	23%	25% ± 5%
Linoleic	53%	55%	50% ± 6%
α-Linolenic	8%	6%	7% ± 2%
Palmitic	11%	12%	11% ± 3%
Stearic	4%	4%	4% ± 2%

For the composition of the intracellular storage, the average and the standard deviation over a dataset of six individual samples are provided. LCFA, long chain fatty acid.

**Figure 5:**
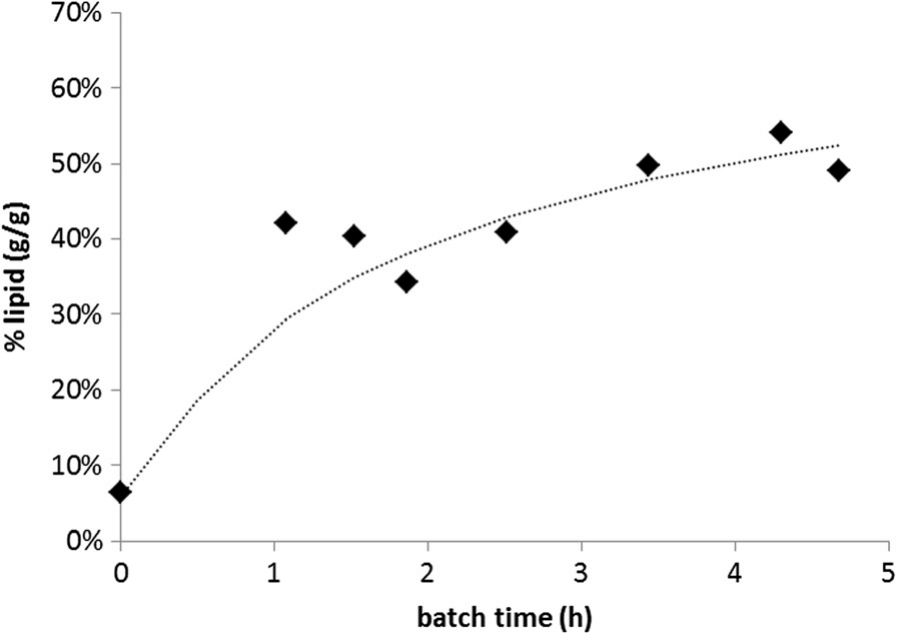
**The evolution of lipid content of the enriched microbial culture over time in a fed-batch experiment with nutrients present.** h, hours.

The maximum lipid storage capacity of the culture was evaluated using fed-batch experiments in which soybean oil was available in excess. The maximum lipid content appeared to be dependent on the availability of nitrogen. In the absence of a nitrogen source, only 24% lipid storage on VSS basis was observed while in another experiment with ammonium present in the reactor liquid(C:N molar ratio was 4/1 Cmol/Nmol), a lipid accumulation of 54% on VSS basis was observed. While the initial lipid accumulation was relatively fast, the lipid content of the culture stabilized after 4 to 5 h of operation, indicating the maximum storage capacity of the culture under these conditions was reached (Figure 5).

### Lipase activity assays

The lipase activity was measured using a standard test with 4-nitrophenyl-palmitate [36] during the feast and the famine phases. An activity of 0.80 ± 0.28 mmol/(l min) was measured during the feast phase (average ± standard deviation over a dataset of six measurements). During the famine phase, an activity of 0.73 ± 0.27 mmol/(l min) was measured (average ± standard deviation over a dataset of three measurements). This indicated that there was no significant difference in lipase activity between the feast and the famine phases. For all cases, we evaluated the activity of both the reactor broth (with biomass) and the reactor supernatant. The average activity measured in the reactor broth was 0.99 ± 0.18 mmol/(l min) and in the supernatant 0.57 ± 0.14 mmol/(l min). This indicated that a significant part of the enzymatic activity was due to extracellular enzymes but also that part of the lipase activity was cell-bound. The measured lipase activity was relatively high compared to the lipid uptake rate observed during the feast phase. Using an uptake of 83 Cmmol (approximately 4 mmol ester bonds) in 2 h and a reactor volume of 2 l, the minimal required lipase activity would be 0.01 to 0.02 mmol/(l min). This indicated that extracellular lipase activity was not limiting the conversion rates.

### Microbial community structure

The molecular and microscopic analyses of the reactor biomass showed a presence of a dimorphic fungus and several bacterial morphotypes. The direct microscopy results were confirmed by fluorescence *in situ* hybridization (FISH) analysis which indicated the presence of both eukaryotes and prokaryotes (Figure 4, left). Furthermore, lipid-specific Nile blue A staining indicated that the fungus was involved in lipid storage while the majority of the prokaryotes in the reactor did not contain significant amounts of lipid-like intracellular storage compounds (Figure 4, right).

**Figure 6:**
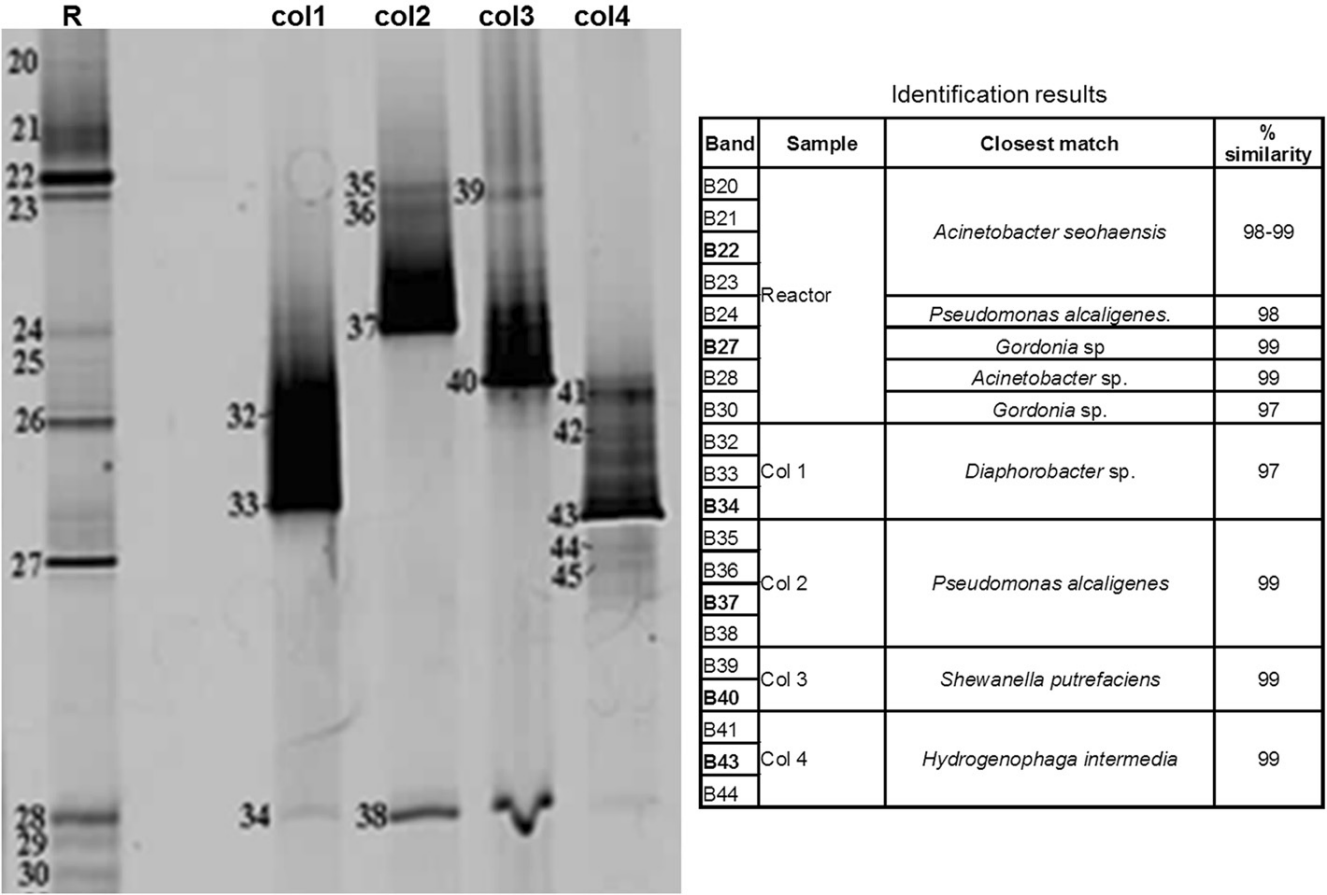
**Bacterial-specific DGGE analysis of the reactor biomass and isolates.** Col 1, LCFA isolate, long rods; Col 2, lipolytic, short motile rods; Col 3, LCFA isolate, small rods, Col 4: glycerol enrichments; DGGE, denaturing gradient gel electrophoresis; LCFA, long chain fatty acid; R, reactor biomass.

Denaturing gradient gel electrophoresis (DGGE) was used to characterize the communities. Eukaryotic primer-based PCR products indicated the presence of *Trichosporon gracile* (100% similarity) from a genus known to include several oleaginous species [27,37]. The bacterial-specific DGGE analysis of the reactor biomass indicated the presence of a few proteobacterial phylotypes closely related to the *Acetinobacter* and *Gordonia* genera (Figure 6), which are both known to possess metabolic pathways for degradation of hydrophobic substrates [38,39].

**Figure 7:**
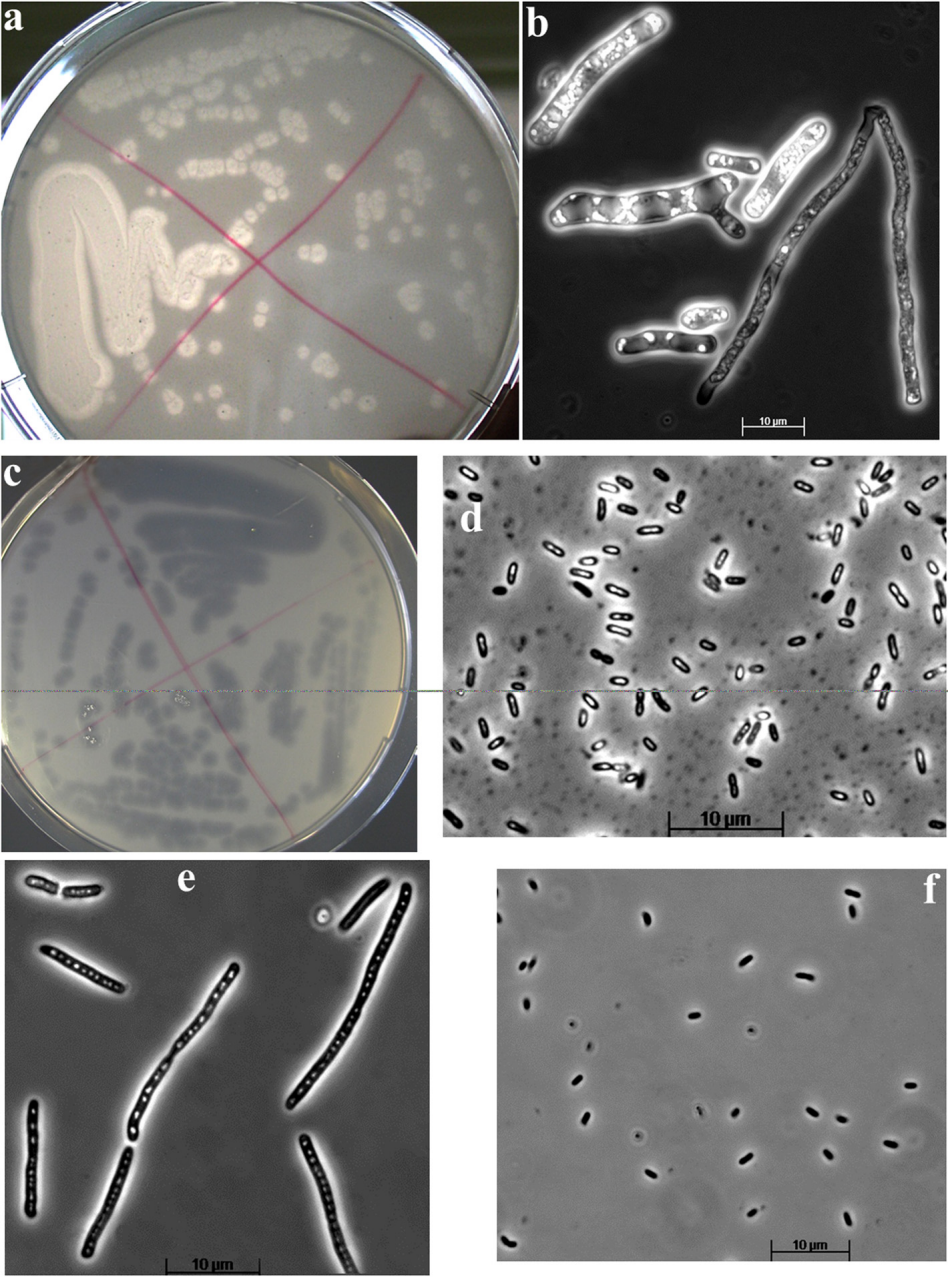
**Different functionalities from isolates obtained by plating of the reactor culture.** Upper panel: *Trichosporon gracile* (**(a)** lypolytic colonies, **(b)** lipid-storing hyphal cells); middle panel: lipolytic *Pseudomonas alcaligenes* (col2) (**(c)** lipolytic colonies, **(d)** cells in liquid culture with lipid); bottom panel: LCFA-utilizing bacterial isolates (**(e)** *Diaphorobacter* sp. (col1) and **(f)** *Hydrogenophaga* sp. (col3)).

Cultivation efforts have been made to try to resolve the role of the microbial species present in the reactor biomass in lipid degradation and utilization of the products. Incubations, with and without antibiotics, allowed the isolation of the eukaryote *Trichosporon* and four prokaryote microorganisms. The eukaryotic dimorphic fungus *T. gracile* was isolated on lipid plates with antibiotics; it formed mycelium-containing colonies surrounded by zones of lipid hydrolysis (Figure 7). This organism was most versatile in its metabolism, being able to degrade the TAG, utilize the products of hydrolysis (LCFA and glycerol), and reform the TAG inside the cells. The bacterial enrichments with three different substrates - lipid, LCFA, and glycerol, yielded four isolates: (i) a small motile rod-shaped bacterium able to grow with lipid and LCFA with strong lipolytic activity (col2, identified as a *Pseudomonas alcaligenes* from the betaproteobacteria). Isolates (ii) and (iii) are two different types of LCFA utilizers. The first LCFA degrader (col1) is a long rod with the ability to store lipids inside the cells. This microorganism was identified as a representative of the genus *Diaphorobacter* from the betaproteobacterial lineage. Although the genus *Diaphorobacter* is quite common for activated sludge, our isolate is very different in cell morphology from the three species described of this genus. The ability to utilize LCFA has never been tested for this genus. The second LCFA degrader is a small rod with no apparent storage polymers (col3) identified as a representative of the genus *Shewanella* in the gammaproteobacteria. (iv) Glycerol-specific enrichment resulted in a domination of *Hydrogenophaga* sp. (betaproteobacterium) represented by small motile rods. Summarizing, the cultivation results showed two functional parts - a eukaryotic dimorphic fungus, doing a complete job of lipid degradation and lipid accumulation, and a bacterial block, basically doing equivalent work but divided between different species (Figure 7).

Despite that two of the bacterial species (*Diaphorobacter* and *Pseudomonas*) were clearly recognizable by microscopic inspection of the reactor biomass, only the latter was detected directly by DGGE analysis of the reactor biomass. One of the possible explanations is a DNA extraction bias in the presence of lipids.

## Discussion

A microbial culture capable of accumulating lipids from a dilute emulsion was enriched in a sequencing batch reactor with a feast-famine regime. The development of this enrichment from an activated sludge inoculum occurred relatively fast (25 days). A lipid-storing fungus and a variety of proteobacterial species co-existed in the enrichment culture. Microscopic observation of the reactor biomass indicated that the bacterial phenotypes (except a small population of large cells identified as a *Diaphorobacter* sp.) did not store significant amounts of lipids but instead used lipids or its immediate hydrolysis products directly as growth substrates.

Analysis of the microbial community by DGGE indicated the presence of the eukaryotic fungus *T. gracile*, which could be readily isolated by microbial plating techniques with antibiotics and appeared to be able to accumulate significant amount of lipids intracellularly. The bacterial population analysis was more complicated since *Gordonia* and *Acinetobacter* species that were detected by DGGE could not be isolated using microbial plating techniques. *Gordonia* and *Acinetobacter* species are important species in the lipid-degrading populations that cause foaming in wastewater treatment plants [40], and especially *Gordonia* species are known to accumulate large amounts of intracellular lipids [41]. Furthermore, while *Diaphorobacter* was observed in the biomass (based on its morphology) and could be readily isolated by microbial plating; it could not be detected by DGGE analysis. This shows that microbial analysis tools such as microbial plating and DGGE can easily introduce bias in the population analysis of a reactor. In conclusion, we could identify and culture the dominant microorganism in the culture, a yeast, but the major bacteria could not be exclusively identified by DGGE or microbial plating.

The presence of the bacterial side population in the reactor may be partly explained by the role of bacteria that by themselves were not able to hydrolyse and use TAG as substrate, but could thrive in the reactor by using the LCFA or glycerol that was liberated through the extracellular lipase activity facilitated by, for example, *Trichosporon.* Furthermore, the coexistence of storing and non-storing microbes indicates that lipid uptake and storage by *T. gracile* was not fast enough to outcompete direct growth by bacterial lipolytic and LCFA utilizers. The specific uptake rate of the microbial culture was with 1 Cmol/(Cmol h) relatively low compared to similar feast-famine systems on other substrates, for example, an uptake rate of around 4 Cmol/(Cmol h) was reported for a feast-famine culture cultivated using acetate as carbon source [42].

The measured hydrolytic activity was more than an order of magnitude higher than the observed uptake rate suggesting that hydrolysis was not the rate limiting step. It should be noted that the values obtained from the enzyme assay only serve as rough indication since the lipase activity may vary when TAG is the substrate instead of 4-nitrophenol-palmitate [36,43]. Factors that potentially limit the lipase activity include transport limitations due to formation of an emulsion during the feast phase [44].

In this study, we show the use of a feast-famine based enrichment strategy for the enrichment of a mixed microbial culture with enhanced lipid accumulation capacity. Remarkably, the maximum accumulation of intracellular lipids of 54% on VSS basis was only achieved when excess nutrients were supplied. This is in contrast with PHA-producing enrichment cultures, which were reported to achieve higher storage contents during nitrogen-deficient fed-batch experiments [45]. Possibly, the major lipid-storing species could not function under nitrogen-limited conditions. Further research may include investigation of the behavior of pure cultures of *T. gracile* to evaluate this hypothesis.

The lipid content of the microbial enrichment culture obtained in this study was around 50% on VSS mass basis and was mainly related to the presence of the oligeanous yeast *T. gracile.* The *Trichosporon* genus is known to be able to accumulate high amount of lipids, for example, TAG contents higher than 60% were reported in pure culture experiments [27]. The high TAG content of these cultures suggest that further improvement may be achieved by development of strategies that provide a competitive advantage for *Trichosporon* over the bacterial side population.

Nevertheless, the achieved lipid content of around 50% is already significantly more than the 10% lipid content in sludge that was used as reference content to evaluate the feasibility biodiesel production [20] or and even more than the oil content of soybeans [46] or olives, implicating the potential feasibility of lipid recovery for the production of biodiesel. Herewith, we show that ecological principles can effectively be employed for the enrichment of lipid-accumulating species, enabling potential application on a broad variety of waste streams. The use of enrichment cultures decreases capital and operating costs because (semi-continuous) open cultivation techniques can be used without the need for sterilization or re-inoculation.

## Conclusion

A microbial enrichment culture of lipid-storing organisms in co-existence with microorganisms that used TAG for direct formation of biomass was observed in a soybean-oil-fed reactor system. The former was represented by a lipolytic dimorphic fungus, while the latter consisted of a consortium of proteobacteria capable of utilizing either the lipid itself or its hydrolysis products, mainly as a substrate for direct growth. The maximum lipid accumulation capacity of the culture was 54% on VSS mass basis. Results were obtained from an enrichment reactor that was not optimized for maximum storage capacity, suggesting that future optimization may lead to microbial enrichment cultures with even higher lipid storage capacity.

## Methods

### Enrichment reactor setup and operational parameters

A SBR with a liquid volume of 2 l was operated aerobically, similar to Johnson *et al*. An operational cycle comprised of a feed phase of 30 min, during which TAG and nutrients were dosed, followed by a reaction phase of 11 h. At the end of the cycle, half of the volume of the reactor was discharged, to be replaced with new feed in the next cycle, resulting in a total cycle length of 12 h, a hydraulic retention time (HRT) and solid retention time (SRT) of 24 h. The reactor was completely mixed (1,000 rpm) throughout the cycle, and biofilms were structurally removed (several times per week) to ensure that the SRT equaled the hydraulic retention time. The temperature was controlled at 30°C ± 1°C using a thermostat, and the pH was maintained at 7.0 ± 0.1 by pH control using 1-M NaOH and 1-M HCl. The gas flow into the reactor was 1.5 l/min consisting of 0.3 l/min air and 1.2 l/min recycled off-gas. The off-gas was cooled to 5°C by a condenser to minimize water evaporation.

### Medium composition

Soybean oil (Markant merk, Beesd, Gelderland, The Netherlands) was chosen as representative model substrate for vegetable oil waste. The substrate soybean oil was analyzed by GC as described in the ‘Sampling and measurement’ section, and the results can be found in the ‘Results’ section (Table 1); the composition was similar to that of soybean oil found in literature [47]. In each cycle, 1.3 g (equivalent to 83 Cmmol) soybean oil was dosed into the reactor using a precision syringe pump. Additionally, 200 ml/cycle nutrient solution containing 3.6 g/l NH_4_Cl, 3.4 g/l KH_2_PO_4_, 1.4 g/l MgSO_4_ · 7H_2_O, 0.54 g/l KCl, 1.0 g/l EDTA Titriplex III (Sigma-Aldrich, St. Louis, MO, USA), 0.033 g/l ZnSO_4_.7H_2_O, 0.024 g/l CoCl_2_.6H_2_O, 0.024 g/l CuSO_4_.5H_2_O, 0.075 g/l FeSO_4_.7H_2_O, 0.017 g/l (NH_4_)_6_Mo_7_O_24_.4H_2_O, 0.110 g/l CaCl_2_.H_2_O according to Vishniac and Santer [48], and 0.050 g/l allylthiourea (to prevent nitrification) was added together with 800 ml/cycle of water.

### Sampling and measurement

The temperature, pH, and off-gas composition in terms of CO_2_ and O_2_ concentrations were monitored online using a standard sensor (Mettler Toledo, Columbus, OH, USA) and an off-gas analyzer (NGA 2000, Rosemount Inc., Shakopee, MN, USA). In addition, samples were taken regularly at the end of the cycle to measure the TSS and the VSS concentrations according to standard methods [49] and the ammonium concentration using spectrophotometric method (LCK-348, Hach-Lange, Düsseldorf, Germany). For evaluation of the carbon balance, the organic carbon content of the organic solids was measured using a colorimetric method (LCK 381, Hach-Lange, Düsseldorf, Germany).

In order to characterize the dynamics during a cycle, samples were taken to measure the intracellular lipid concentrations, TSS and VSS production, ammonium uptake, CO_2_ production, and O_2_ consumption. Intracellular storage compounds were analyzed as described by Johnson *et al*. [6] except that a modified gas chromatography method was used: analysis of extracted PHA and lipids derivatives was performed using direct injection into a polyethylene glycol column (HP-INNOWax, 60 m × 250 × 0.15 μm) installed on a gas chromatograph (model 6890 N, Agilent Technologies, Santa Clara, CA, USA) equipped with a flame ionization detector. The column temperature program comprised an initial oven temperate of 100°C, operated for 2 min, followed by an increase to 170°C at a rate of 20°C/min, and a further increase to 240°C at a rate of 5°C/min resulting in a total runtime of 30 min per sample. The temperatures of the injector and detector were 230°C and 250°C, respectively. Calibration standards included PHB, PHV, hydroxyhexanoate, and hydroxypalmitate for identification of PHA, and oleic-, linoleic-, a-linolenic-, palmitic-, and stearic-acid for identification of LCFAs. For each standard, three different concentrations were used to make a calibration curve. For each GC spectrum, it was checked whether there were unknown peaks indicating missing compounds.

The lipase activity in the culture was evaluated using a colorimetric method based on the hydrolysis of an artificial lipid, 4-nitrophenyl-palmitate [36] both in untreated samples and in samples in which biomass had been removed by centrifugation for 10 min at 2,500 rpm. The hydrolysis product, 4-nitrophenyl was measured spectrometrically at 410 nm.

### Microbial community structure

The general microbial community composition was investigated using FISH analysis as described by Johnson *et al*. [6], using the EUB338 and EUK516 probe mixtures for identification of bacterial and eukaryote species, respectively [50]. Intracellular storage compounds were visualized using Nile blue A staining. The microbial diversity was further analyzed by DGGE using eukaryotic and prokaryotic primers [51]. Additionally, traditional cultivation techniques were used to separate the functional groups of microorganisms from the reactor and to isolate pure cultures. For this, serial dilutions in liquid medium containing three different substrates were used: soybean oil, the free LCFA fraction of soybean oil, and glycerol. To separate the eukaryotic community from bacteria, a mixture of kanamycine and streptomycine (100 mg/l each) was applied in a dilution series. The final positive dilutions on each substrate were plated on solid media with corresponding substrates, except that LCFA and soybean oil were first emulsified by sonication with gum arabic to allow detection of the LCFA-utilizing and lipolytic colonies [52].

### Fed-batch accumulation experiments

The storage capacity of the microbial enrichment culture was assessed using a fed-batch accumulation experiment. Apart from the feeding regime, all other operational parameters were kept identical to the enrichment reactor: pH was maintained at 7.0 ± 0.1, the temperature was maintained at 30°C ± 1°C, and the reactor liquid was stirred with 1,000 rpm. The biomass from the enrichment reactor was used as inoculum, and the oxygen profile was used to monitor the activity in the reactor; when the oxygen consumption decreased, a new pulse of soybean oil substrate was dosed. The biomass was characterized in terms of lipid content over time.

### Data analysis and interpretation

It was not possible to take a representative sample for measurement of the substrate concentration from the oil-water emulsion that formed quickly after substrate dosage. Instead the soybean oil uptake rate in the medium was estimated by assuming that all the oil had been taken up after the feast phase. This assumption was verified using microscopy to make sure that all oil had been taken up and was not absorbed to the biomass. The length of the feast phase was estimated using the off-gas data, with the feast phase characterized by relatively high oxygen consumption and CO_2_ production rates and the onset of the famine phase by a relatively sharp decline in both these rates (Additional file 1: Appendix A). The active biomass concentration was calculated by subtracting the amount of storage compounds from the amount of VSS in the system. Active biomass concentrations were verified by the evaluation of the ammonium uptake and nitrogen content of the biomass. Conversion yields and rates were obtained using a modified version of a model used for modeling PHA-producing feast-famine cultures [53]. The only modifications in the model were replacement of PHA storage by storage of TAG and inclusion of a fraction of the substrate that was used for direct growth. A description of the model is provided in the (Additional file 1: Appendix C).

## Additional file

Additional file 1:
**Appendix A: Off-gas profile.** Appendix B: Carbon flow sheet. Appendix C: Model.

## Abbreviations

GC: gas chromatography
HRT: hydraulic retention time
LCFA: long chain fatty acid
PHA: polyhydroxyalkanoate
SBR: sequenced batch reactor
SRT: solid retention time
TAG: triacylglyceride
TSS: total suspended solids
VFA: volatile fatty acid
VSS: volatile suspended solids
