# Supplementary material for: Lipid recovery from a vegetable oil emulsion using microbial enrichment cultures
Source: Biotechnol Biofuels. 2015 Mar 10;8:39. doi: 10.1186/s13068-015-0228-9 (PMC4369065; doi:10.1186/s13068-015-0228-9)
Supplement: Additional file 1: — Appendix A: Off-gas profile. Appendix B: Carbon flow sheet. Appendix C: Model. [file 13068_2015_228_MOESM1_ESM.docx]

**Appendix A – off-gas profile**

Off-gas measurements showed decreasing oxygen and increasing CO_2_ concentrations in the first part of the cycle, indicating increased activity defined here as the feast phase, followed by a relatively sharp decline indicating the start of the famine phase (figure A1).

**
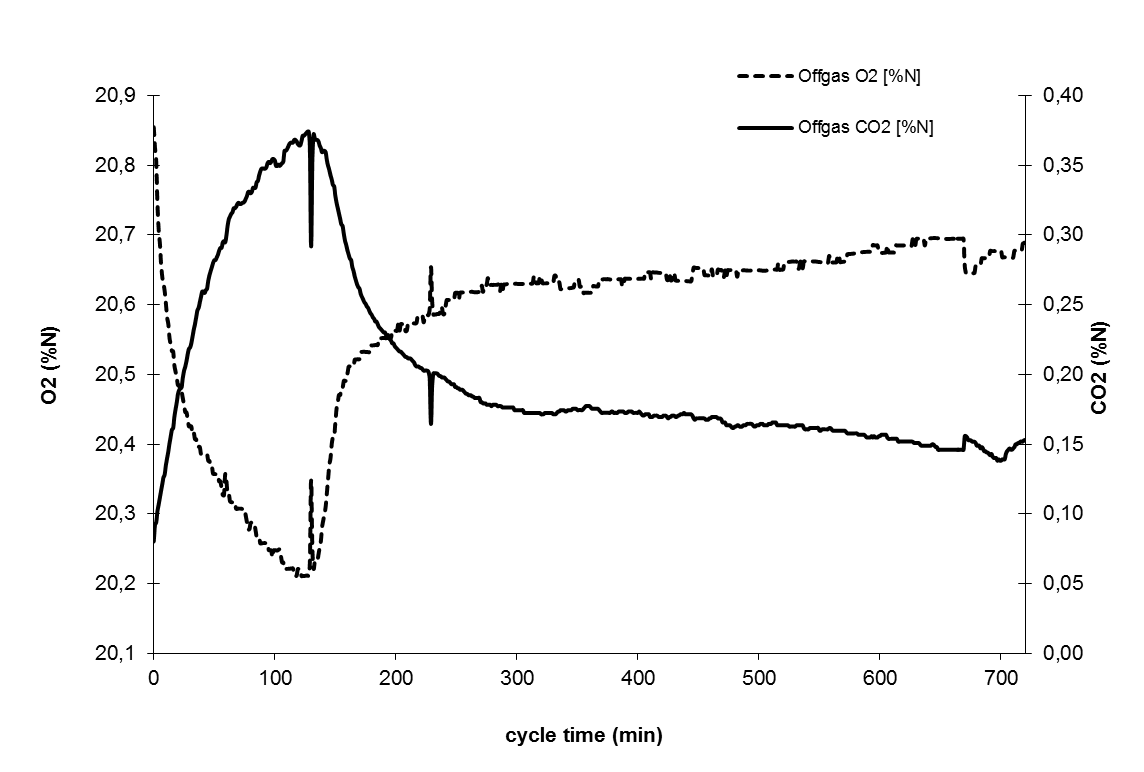
**

Figure A1: Typical off-gas profile of the reactor, indicating an increased activity after dosing a pulse of vegetable oil at the start of the cycle and a relatively sharp decline of activity after around 120 minutes. The small peaks around 120 and 230 minutes were caused by sampling.

**Appendix B – Carbon flow sheet**

Figure B1: Carbon balances of the enrichment reactor during steady operation.

**Appendix C - Model**

General

The model structure was derived from the metabolic model used to model PHA accumulating microbial enrichment cultures [ref]. An explanation for the rationale behind the used kinetic equation can be found in the latter article. Here we describe the most important kinetic equations and the model calibration.

Kinetic equations

Uptake rate kinetics were described by a Monod equation

$$q_{S}=q_{S}^{\max}\frac{C_{S}}{C_{S}+K_{S}}$$

The intracellular lipid conversion rate was calculated by subtracting the consumption rate from the production rate.

$$q_{lipid}=Y_{lipid,S}\cdot q_{S}-k\cdot\left(\frac{C_{X0}}{C_{X}}\right)^{1/3}f_{lipid}^{2/3}$$

The growth rate (µ) was established as the resultant of

1. Direct growth on external substrate
2. Growth intracellular substrate

$$\mu=\left(q_{S}\cdot(1-\alpha_{storage})+k\cdot\left(\frac{C_{X0}}{C_{X}}\right)^{1/3}f_{lipid}^{2/3}\right)\cdot Y_{X,lipid}$$

The nitrogen consumption rate was stoichiometrically coupled to the growth rate using

$$q_{N}=0.2\mu$$

Model calibration

For each sampling time point the modeled data for each compound was compared with the measured data. The errors were calculated, squared, and summed up as follows

$$SSE=\sum_{i=1}^{N} (n^{measure}(t_{i})-n^{model}(t_{i}))^{2}$$

The sum of squared errors for the different compounds were summed up to the total error between model and measurements (*E_M_*).

$$E_{M}=\sum SSE_{i}$$

with *i* = S, lipid, X, N

The total error was minimized by adjusting the parameter values for the yields (Y_lipid,S_ and Y_X,lipid_), the fraction of the substrate converted to storage lipids (α) and the biomass specific uptake rate (q_S_). The substrate affinity constant was assumed not be be relevant for the system and was set to 2 Cmol/l, effectively minimizing its influence. Minimization was performed by the solver tool of Microsoft Excel. Carbon and COD balances were not included as criteria for model calibration and were evaluated separately.

Nomenclature

C_S_ concentration of substrate [Cmol/l]

C_X_ concentration of biomass [Cmol/l]

C_X0_ initial concentration of active biomass [Cmol/l]

f_lipid_ ratio of PHA to active biomass [Cmol/Cmol]

k rate constant for shrinking particle model [Cmol lipid^1/3^/(Cmol X^1/3^·h)]

K_S_ substrate affinity constant [Cmol/l]

q_S_ biomass specific substrate uptake rate [Cmol/(Cmol·h)]

q_S_^max^ maximum biomass specific substrate uptake rate [Cmol/(Cmol·h)]

Y_i,j_ yield of compound i on compound j [Cmol/Cmol]

α fraction of the substrate converted to storage

µ biomass specific growth rate [Cmol/(Cmol·h)]
